# Comparing the Efficacy of SNP Filtering Methods for Identifying a Single Causal SNP in a Known Association Region

**DOI:** 10.1111/ahg.12043

**Published:** 2013-11-11

**Authors:** Amy Victoria Spencer, Angela Cox, Kevin Walters

**Affiliations:** 1 School of Mathematics and Statistics, University of SheffieldSheffield, S3 7RH, UK; 2 CRUK/YCR Sheffield Cancer Research Centre, Department of Oncology, University of SheffieldSheffield, S10 2RX, UK

**Keywords:** Fine-mapping, likelihood, single nucleotide polymorphism, complex disease, causal variants, LD, *p*-value

## Abstract

Genome-wide association studies have successfully identified associations between common diseases and a large number of single nucleotide polymorphisms (SNPs) across the genome. We investigate the effectiveness of several statistics, including *p*-values, likelihoods, genetic map distance and linkage disequilibrium between SNPs, in filtering SNPs in several disease-associated regions. We use simulated data to compare the efficacy of filters with different sample sizes and for causal SNPs with different minor allele frequencies (MAFs) and effect sizes, focusing on the small effect sizes and MAFs likely to represent the majority of unidentified causal SNPs. In our analyses, of all the methods investigated, filtering on the ranked likelihoods consistently retains the true causal SNP with the highest probability for a given false positive rate. This was the case for all the local linkage disequilibrium patterns investigated. Our results indicate that when using this method to retain only the top 5% of SNPs, even a causal SNP with an odds ratio of 1.1 and MAF of 0.08 can be retained with a probability exceeding 0.9 using an overall sample size of 50,000.

## Introduction

Genome-wide association studies (GWAS) and candidate gene studies have highlighted regions of the genome containing variants affecting disease susceptibility. The next stage is fine-mapping of these regions to identify the variants most likely to be causal. This task is confounded by high correlation between variants in a small chromosomal region. The effects of this correlation as well as sampling variation mean that in tests of association the variant with the largest likelihood or smallest *p*-value will not necessarily be the causal variant. Several statistical methods for analysing fine-mapped data have now been published but guidelines are needed to determine which of these will give the highest true positive rates (TPRs) and lowest false positive rates (FPRs) and in which scenarios.

Methods for analysing fine-mapped data include those that analyse multiple variants in a region simultaneously, for example, penalised and nonpenalised regression methods and Markov chain Monte Carlo routines. Some such methods are given in reviews by Ayers & Cordell (2010) and Abraham et al. (2013), including the popular HyperLasso (Hoggart et al., 2008). There are also fully Bayesian methods implemented in the software pi-MASS (Guan & Stephens, 2011). Also, some recent methods attempt to include external data such as functional annotation, for example, *p*-value weighting (Saccone et al., 2008) and a Bayesian latent variable model (BLVM, Fridley et al., 2011). However, we have chosen to compare a subset of statistical analyses which should work well when a single causal variant is present in the chromosomal region of interest. In these methods, each single nucleotide polymorphism (SNP) is analysed separately and they are then ranked in some way based on the likelihood or *p*-value from a logistic model or based on linkage disequilibrium (LD) with or proximity to the top hit SNP in the region. The methods we consider do not make use of any available functional data. To our knowledge this set of methods has not previously been compared in a thorough simulation study such as this.

All of the statistics that this report examines could be used as filters to remove noncausal variants from the set of all candidate causal variants. The variants considered in this work are SNPs but the methods and results discussed can be applied directly to any other variants which can be modelled via a logistic regression model. Successful filters will reduce the initial set of SNPs down to a much smaller group in which it is highly probable that the true causal variant remains. Other techniques, such as the biological analysis of pathways in cell lines, can then be used to identify the causal variant. These methods are expensive, so reducing the number of variants to take forward is of paramount importance.

The first methods we examine are based on *p*-values and likelihoods. It is common in GWAS to rank SNPs by *p*-values either from Cochran–Armitage trend tests or from Wald tests and both of these methods have now also been used in the context of fine-mapping (Miki et al., 2010; Adrianto et al., 2012). An alternative to using *p*-values is to use the likelihood (or equivalently log-likelihood) from fitted regression models. Several studies (including Easton et al., 2007; Udler et al., 2009, 2010a; French et al., 2013), rank SNPs based on likelihoods and the usual practice is to retain the set of SNPs with likelihoods within a prespecified ratio of the highest likelihood. This method leads to variable numbers of SNPs being retained. We examine this relative likelihood (RL) filter as well as the alternative of retaining a prespecified proportion of all SNPs based on ranking by likelihood. These statistics are attractive for filtering because they are easily obtained from standard analyses.

The remaining methods relate to LD structure. Within a small chromosomal region, LD can be high between SNPs. When the top hits from GWAS are found, these are not assumed to be the causal SNPs, but it is often postulated that the causal SNP lies within the same gene or LD block as the tagSNP. Alternatively, a handful of candidates may be suggested based on high LD with the tagSNP (*r*^2^ > 0.9, for example). We formalise three filtering methods based on these ideas: ranking by genetic map distance, *r*^2^ and *D*′ with the top hit (the SNP with the largest likelihood). The final method (Zhu et al., 2012) we examine is also LD-based, but takes into account the LD between each SNP and the top hit compared to the LD between the SNP and tagSNPs in the region. Although we use the analyses set out by Zhu et al. (2012), we use it in a slightly different setting, as it is designed for use with tagSNPs from a GWAS. As far as we are aware the application of these LD- and distance-based methods to fine-mapped genotype data and their comparison with standard univariate statistical methods is novel.

We found that percentile filtering based on ranked likelihoods was the most efficacious method in all the scenarios we investigated. To explore the utility of this approach, this study considers the impact of effect size, sample size, minor allele frequency (MAF), mode of inheritance and filter threshold on the effectiveness of the filter proposed. We also consider whether these results apply to filtering in regions of the genome with strikingly different LD structures. A range of plausible odds ratios (ORs) were used in our simulations, as well as relatively large sample sizes consistent with numbers being used in the era of disease-specific consortia.

## Materials and Methods

### Simulation Details and Preliminary Analysis

Filters were tested by applying them to simulated genotype data with a single causal SNP. Causal SNPs were chosen based on their MAF and results were examined for scenarios with different causal SNPs, ORs and sample sizes. By simulating data with a known “true” causal SNP, it was possible to determine whether or not this SNP was retained in the set of all candidate causal SNPs after filtering. All datasets mentioned were simulated using the hapgen2 software (Spencer et al., 2009). The software generates haplotype sequences based on MAF and LD structure in a reference dataset, in this case the European haplotypes of the August 2010 release of the 1000 genomes data (The 1000 Genomes Project Consortium, 2010), and a user-specified effect size for the causal SNP.

We chose three regions of the genome to test the methods on. Several studies have found evidence to suggest that the region around the *CASP8* gene on Chromosome 2 (a gene which codes for a protein involved in apoptosis) may include variants which affect the risk of developing breast cancer and more recently melanoma (Cox et al., 2007; Han et al., 2008; Palanca Suela et al., 2010; Barrett et al., 2011; Camp et al., 2012). A one megabase region (from 201,566,128 to 202,566,128 bases in the Hg19 build of Chromosome 2) containing *CASP8* was used for simulations. This region also contains around 20 other known genes including *CASP8* homologues *CFLAR*, *CASP10* and several *ALS2CR* genes. In this 1 Mb region, there were 2871 SNPs in the August 2010 1000 genomes data (The 1000 Genomes Project Consortium, 2010). This region has mixed LD block sizes averaging approximately 22 kb in length, so for comparison, two other regions were selected which have particularly high and particularly low levels of LD. Using results in Smith et al. (2005), we carefully selected a region of Chromosome 11 (55–56 Mb, part of the *MHC* region, average LD block size ≈130 kb), and a region in Chromosome 16p13 (9–10 Mb, average LD block size ≈8 kb). These 1 Mb regions contained 6247 and 6200 SNPs, respectively (1000 genomes, August 2010).

We focused on additive models, varying the per-allele OR of the causal SNP between 1.06 and 1.24, but other modes of inheritance were also considered. The causal SNP was also varied, with MAFs between 0.08 and 0.31, as well as the sample size between 10,000 and 50,000. The sample sizes quoted in this paper represent the total number of cases and controls, which are always assumed to be equal. We refer to a specific causal SNP, OR and sample size as a “scenario” and for each scenario simulate a large number of datasets (usually 1000). The results from the analysis of all 1000 datasets were then used to assess the filters.

For each SNP, a univariate logistic regression model is fitted (one SNP per model) so that we are only considering marginal effects. For SNP *i*, the probability that a subject *j*, with *x*_*ij*_ copies of the allele coded “1,” has the disease is *y*_*ij*_ and is given by
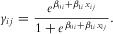
1
*β*_1*i*_ is the per-allele log odds ratio (logOR) of disease for the allele coded “1” compared to the allele coded “0” for that SNP. R (R Core Team, 2012) was used to fit the logistic regression models and to obtain the maximum likelihood estimates (MLEs) of *β*_0*i*_ and *β*_1*i*_, the likelihood of the parameters for SNP *i*, denoted 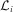 and the *p*-values from Cochran–Armitage and Wald tests.

### Filters Based on *p*-Values and Likelihood

All the methods that we compare filter out SNPs from the set of all candidate causal variants to leave a smaller subset. For each method, the chosen filtering statistic is calculated for each variant and a threshold is applied. The first two filtering statistics are the *p*-values from Cochran–Armitage tests and those from Wald tests, and a threshold value may be chosen based on a Bonferroni correction, for example. Although we carried out filtering using both *p*-value methods, the results were always very close, so we consider these as equivalent methods from now on and report just one, labelling it the *p*-value method.

The RL for the *i*th SNP compares the maximised likelihood for SNP *i* to the largest of the maximised likelihoods over all *p* SNPs in the region:
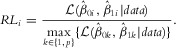
2These RLs can range from close to zero to one (for the SNP which satisfies the denominator and which we call the “top hit” or SNP_*max*_). In the papers by Udler et al. (2009, 2010a), the RL filter threshold of 1/100 was generally used, filtering out all SNPs with an RL <1/100. We also briefly examine the use of different thresholds for RL filtering.

A possible weakness of RL filtering is that the number of SNPs retained is subject to variation. An alternative is to rank the likelihood values for each SNP and filter out a prespecified number or proportion of SNPs. This filter is called the likelihood percentile (LP) filter and by definition it is known how many SNPs will be retained (for example, a threshold of 95% retains the top ranked 5% of SNPs). This approach has the potential advantage that it will not be affected by a single extreme likelihood value at one particular SNP due to sampling variation.

### Filters Based on Genetic Map Distance or LD between Variants

The remaining filters that we investigated also relate individual SNPs to SNP_*max*_. These methods of filtering are based on the principle that while SNP_*max*_ may not itself be causal, the true causal SNP is likely to be “close to it” in some sense, either physically close or highly correlated with it. For three of the methods, SNPs were ranked by either genetic map distance in centimorgans (cMs) from SNP_*max*_ or by pairwise *D*′ or *r*^2^ values with SNP_*max*_. Genetic map distances were obtained from the 1000 genomes data (The 1000 Genomes Project Consortium, 2010) and pairwise LD (*D*′ and *r*^2^) values were calculated using the simulated haplotypes. Once again, thresholds were specified so that SNPs further away in distance or with lower LD values than those thresholds were filtered out.

The final filtering method (Zhu et al., 2012) was also based on *r*^2^ between each SNP and SNP_*max*_, but rather than ranking based on this value alone, a preferential LD (PLD) score was calculated for SNP_*i*_. This method is designed for use with GWAS data so makes use of the panel of tagSNPs from the genotyping array. PLD_*i*_ is the proportion of tagSNPs for which *r*^2^ between them and SNP_*i*_ is greater than between SNP_*max*_ and SNP_*i*_. For the simulated regions, since all SNPs have been “genotyped,” we chose to use those on the Illumina 300 array as our tagSNPs. There were 77 such SNPs in both the *CASP8* and *MHC* (mixed and high LD) regions and 135 in the 16q13 (low LD) region. To complete the Zhu method, a second filtering step is required, which involves calculating an empirical *p*-value testing the *r*^2^ value between SNP_*i*_. Specifically, this *p*-value “estimates the probability of observing the same or better *r*^2^ value for two random variants with the same frequencies” (Zhu et al., 2012). This is done by permuting the genotypes 2000 times in each dataset. This number of permutations was too computationally expensive when analysing 1000 simulated datasets, so the Zhu method was only tested on a subset of 100 datasets for each scenario.

### Robustness of Filters When Imputation is Used

Imputation of SNPs which are not genotyped is now common, as it is still too costly to genotype every SNP and methods of imputation based on MAF and LD have been shown to be reliable. To test how well these filtering methods work when some SNPs are imputed compared to when they are all genotyped, we simulated several sets of 100 datasets, covering various causal SNP scenarios within the *CASP8* region. To test the effect of imputation, we then chose a list of 469 informative SNPs to keep as “genotyped,” based on prior knowledge of the region, as would happen in the planning stages of a fine-mapping project. All other SNPs were removed, which always included the causal SNP. The missing SNPs were then imputed using the software impute2 (Marchini & Howie, 2010) and the data reanalysed. The results of analyses of the fully genotyped and the partially imputed datasets could then easily be compared.

## Results

### Receiver Operating Characteristic (ROC) Curves

We have used ROC curves to display the results of filtering on different datasets. For each scenario (fixed causal SNP, effect size and sample size), multiple datasets were simulated to allow for sampling variation. The mean FPR is given on the *x*-axis of each ROC curve, and this refers to the mean proportion of noncausal SNPs retained over all of the simulated datasets. The TPR plotted against this on the *y*-axis is the probability of the true causal SNP being retained at the corresponding thresholds, calculated as the proportion of the simulated datasets in which the causal SNP was retained. The TPR and FPR when filtering at specific thresholds of interest are highlighted using points on the ROC curves.

We believe these are appropriate summary statistics for the results of the simulation analyses, but it should be noted that there is no single, standard method of combining the results of multiple tests into a single ROC curve. This is discussed in detail in a paper by Fawcett (2006), in which the author describes three possible methods for creating such an ROC curve. The way we have calculated TPRs and mean FPRs is equivalent to the method that Fawcett (2006) calls “threshold averaging” and it results in variation around the curve in both dimensions. The variation around the mean FPR is given by the range of FPR values from all simulations. TPR is a sample proportion from a binomial distribution, so the variance can be calculated using TPR(1 − TPR)/*n*, where *n* is the number of simulations.

### Relative Efficacy of Different Filtering Methods

**Figure 1:**
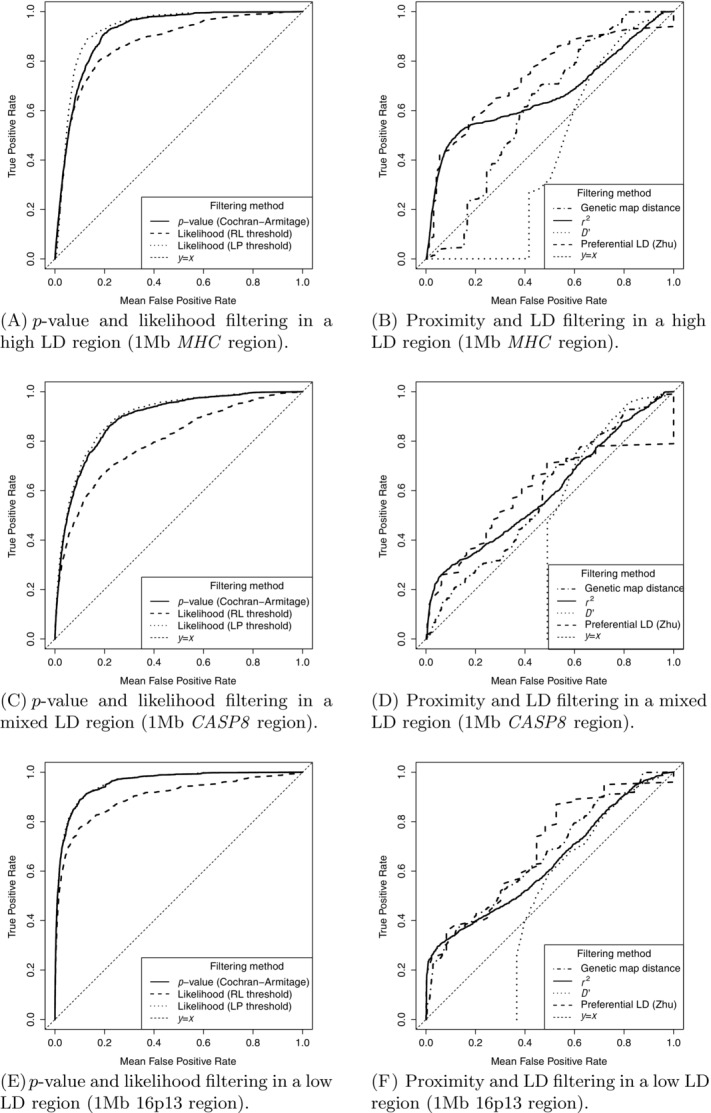
Comparing the effectiveness of filters for fine-mapped data in three regions of the genome. Using the LD structure of each region, 1000 datasets were simulated and then analysed using each method (only 100 were analysed using the Zhu method). Panels (A), (C) and (E) show the efficacy of filtering using thresholds based on *p*-values from Cochran–Armitage tests, RLs and LP points. Panels (B), (D) and (F) show the results using genetic map distance (GMD) from and pairwise *r*^2^ or *D*′ values with the top hit and the Zhu method using preferential *r*^2^. The causal SNPs all have an OR of 1.1, an MAF of 0.08 and the sample size is 20,000.

Figures 1(A) and (B) show ROC curves for the different filtering methods used on the same set of 1000 datasets for fine-mapping the high LD *MHC* region. These simulations use a sample size of 20,000 and have a causal SNP with an OR of 1.1 and MAF of 0.08. Figure 1(A) shows the results from the *p*-value and likelihood-based methods. Figure 1(B) compares the efficacy of the proximity and LD-based methods. It should be noted that for computational reasons the Zhu (PLD) filtering method was only carried out on a subset of 100 of the simulated datasets. Figures 1(C)–(F) display the equivalent outcomes of filtering in the mixed LD (*CASP8*) and low LD (16q13) simulated datasets. Table1 contains the area under the curve (AUC) values as percentages of the total possible area for all of the ROC curves in Figure 1, and Table2 gives the AUCs for the parts of the ROC curves which result in mean FPRs of 0.1 or lower, as these are the parts of the ROC curves that are most of interest. It should be noted that the maximum possible partial AUC as given in Table2 is 10%.

**Table 1:** Area under curve (AUC, given as a percentage) for ROC curves of different filters using mean false positive rates (FPRs). Three different 1 Mb regions of the genome were used but in each the causal SNP has an OR of 1.1, an MAF of 0.08 and the sample size is 20,000

	Genomic region		
	High	Mixed	Low
Filtering method	LD (%)	LD (%)	LD (%)
Likelihood (LP threshold)	93	90	96
*p*-Value	91	89	96
Likelihood (RL threshold)	87	79	90
Preferential LD (Zhu)	74	60	69
*r*^2^	67	60	63
Genetic map distance (GMD)	62	58	66
*D*′	42	42	48

**Table 2:** Area under curve (AUC, given as a percentage) for portions of ROC curves of different filters for which FPR ≤0.1. Three different 1 Mb regions of the genome were used but in each the causal SNP has an OR of 1.1, an MAF of 0.08 and the sample size is 20,000. The maximum percentage of AUC for such a portion is 10%

	Genomic region		
	High	Mixed	Low
Filtering method	LD (%)	LD (%)	LD(%)
Likelihood (LP threshold)	4.8	4.7	7.2
*p*-Value	4.3	4.5	7.2
Likelihood (RL threshold)	4.1	3.6	6.2
Preferential LD (Zhu)	2.5	1.0	2.1
*r*^2^	2.9	2.1	2.8
Genetic map distance (GMD)	0.2	1.0	2.2
*D*′	0.02	0	0

Although these three regions were carefully chosen so that their LD structures were all very different it can clearly be seen that the likelihood and *p*-value-based methods are generally more efficacious than the methods which filter based on proximity to, and LD with, SNP_*max*_ for these scenarios in all three regions. The likelihood method using LP thresholds resulted in the ROC curves with the highest AUCs, with the AUC for *p*-value filtering only slightly lower. So if *p*-values were more readily available, it would be acceptable to use them for filtering. Interestingly we found that in general, larger sample sizes resulted in a bigger difference between the AUCs of the LP and *p*-value methods.

Of the LD- and proximity-based methods, the Zhu method had the highest AUC over the entire FPR range but *r*^2^ was better over the more relevant range of FPRs of 0.1 and under. In all three regions, RL filtering was considerably worse than LP filtering for the single sample size, causal SNP OR and MAF we considered in Figure 1. However, we also examined other scenarios (see the ranges specified in the Methods section) and found that the relative performance of the different filters seem to apply generally for these scenarios as well. Since LP filtering appears to be the best performing filter we now examine its performance in more detail.

### The Effect of Sample Size, the Causal SNP OR and MAF on Results of LP Filtering

**Figure 2:**
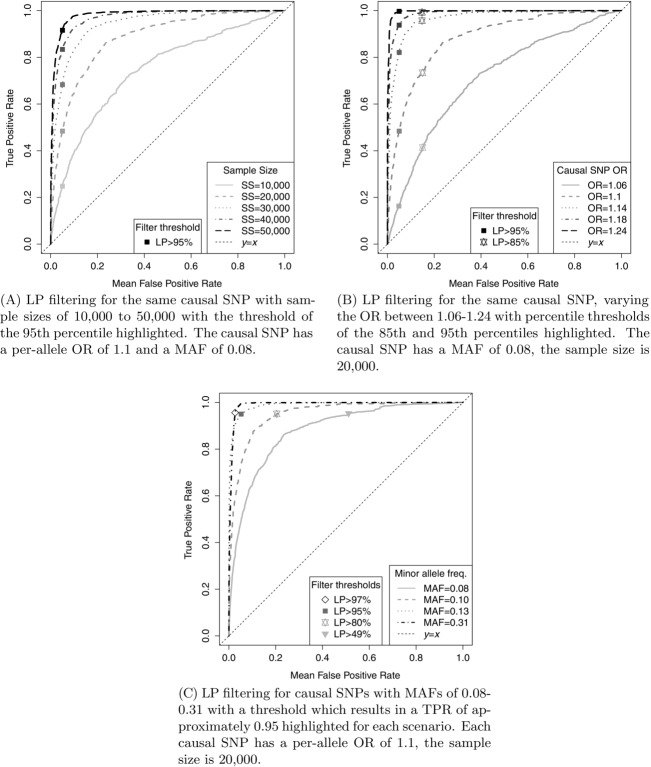
Receiver operating characteristic (ROC) curves showing the effectiveness of likelihood percentile (LP) as a fine-mapping filter dependent on the sample size used, the per-allele OR and MAF of the causal SNP. One thousand datasets were simulated for each scenario using the LD structure of the *CASP8* region and the results of filtering at specific thresholds are highlighted.

Figure 2 shows how the results of LP filtering vary dependent on the sample size, OR and MAF of the causal SNP for the *CASP8* data. Similar results were recorded in the other regions (data not shown). With LP filtering, we fix the total proportion of SNPs retained, and as there is only one causal SNP, this proportion is almost identical to the FPR. Figure 2(A) shows that if there is a fixed proportion of SNPs that can be taken forward (due to experimental costs, for example), then, as expected, the TPR increases as sample size increases. This is also the case as causal SNP OR and MAF increase. Figure 2(B) shows that if a particular FPR does not yield a high enough TPR, then the filter threshold could be relaxed from the 95th to the 85th percentile, say. It is perhaps more relevant to focus on what threshold is required to achieve a particular TPR, so the thresholds given in Figure 2(C) are those that result in a TPR ≥0.95. We focus on these thresholds as we examine the separate plots in more detail.

Figure 2(A) shows how sample size affects LP filter efficacy. For a scenario with a sample size of 10,000 where the causal SNP has an OR of 1.1 and MAF of 0.08, to achieve a TPR of 0.95 a threshold of 15% would be required, meaning that 85% of the SNPs would be retained. At the same TPR, increasing the sample size to 20,000 requires a threshold of 49%. For sample sizes of 30,000, 40,000 and 50,000, the corresponding thresholds are 75%, 86% and 93%. So for a causal SNP with this OR and MAF, sample sizes above 50,000 are required to be 95% sure of capturing the causal SNP while taking forward 5% or less of the original SNPs.

Figure 2(B) shows the results of applying LP filtering as the OR of the causal SNP varies. In the simulations, the sample size was 20,000, the causal SNP had an MAF of 0.08 and the per-allele ORs took values between 1.06 and 1.24. The general increase in AUC with causal SNP OR is clear. At very small ORs such as 1.06, LP filtering requires the majority of the SNPs to be retained in order to achieve a high TPR. For example, for a TPR of 0.9, a filtering threshold of 27% is required and for a TPR of 0.95, a threshold of 14% is required (retaining approximately 2469 SNPs of the 2871 in this dataset). However, to achieve these same TPRs when the OR is 1.14 thresholds of 93% and 87% can be applied. Even for a sample size as large as 20,000, rarer causal SNPs with an OR of 1.1 or less cannot be captured at a TPR exceeding 0.95 without capturing more than half of all SNPs in the region.

Although the results are not given here, we also investigated the utility of filtering for SNPs with different modes of inheritance and found the results to be consistent with those we modelled additively using per-allele ORs.

Figure 2(C) shows the results of SNP filtration with a sample size of 20,000 for different MAFs. Causal SNPs were chosen that had four different MAFs but were located close together in a single LD block within the 1 Mb region simulated (to reduce the possible effects of LD structure). It can be clearly seen from Figure 2(C) that increasing the MAF of the causal SNP from 0.08 to 0.10 increases the AUC of the ROC curve (from 88% to 95%). Further increases in MAF also increase the AUC, although increases above 0.13 (with an AUC of 99%) only lead to negligible improvements in AUC. In this figure, a point is marked on each ROC curve at the threshold which results in a TPR of 0.95. It can be seen that they are 49%, 80%, 95% and 97% when the causal SNP has MAF 0.08, 0.1, 0.13 and 0.31, respectively. With a sample size of 20,000, a causal SNP with an OR of 1.1 would require an MAF greater than 0.1 in order to reduce the set of candidate SNPs to less than 20% of its original size while being 95% sure of capturing it.

### RL Filtering

Previous studies (Easton et al., 2007; Udler et al., 2009, 2010a; French et al., 2013) used RL filtering for fine-mapping, but we have shown that simpler LP filtering results in ROC curves with larger AUCs (illustrated in Fig. 1) in the scenarios we considered. A disadvantage to RL filtering is the large amount of variation between simulated datasets in the FPR using a specified RL threshold. For example, we examined filtering on the 1000 *CASP8* simulations with a sample size of 20,000 and a causal SNP with an OR of 1.1 and an MAF of 0.08 at the threshold used in these studies of 1/100. This results in a TPR of 0.682 across the 1000 datasets. The median FPR across the datasets is 0.105 but the interquartile (IQ) range of the FPR is (0.046, 0.268) and the full range is (0.0003,1), indicating that between 1 and all 2871 of the 2871 original SNPs were retained in the simulated datasets using RL ≥1/100. The full range is still between 2 and 2871 SNPs even at a much more relaxed threshold of RL=1/1000 (TPR=0.962).

We observed that the range of FPRs decreases for RL filtering as the OR increases. A per-allele OR of 1.24 is similar to the estimated effect sizes of the causal SNPs in the studies which have used this type of filtering before (Easton et al., 2007; Udler et al., 2009, 2010a). The sample size of 20,000 in the simulated datasets is also commensurate with their sample sizes. The results for RL filtering for this scenario are not shown, but the AUC (with mean FPR) is very close to 1 and there is very little variability in FPR, suggesting that in general RL filtering was a suitable method to use in these studies. In particular, the mean FPR and TPR at a threshold of 1/100 are 0.031 and 0.987, respectively. The variability between simulations is a clear limitation of RL filtering and we recommend filtering based on likelihood but using a percentile threshold, particularly for OR of 1.1 or less.

### LP Filtering with Imputed Data

**Figure 3:**
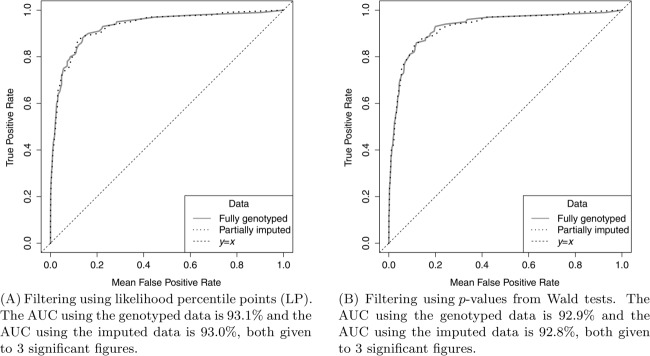
The effectiveness of LP and *p*-value filtering for fine-mapping data which has been partially imputed compared to its effectiveness for data which is fully genotyped. The causal SNP has an OR of 1.14, an MAF of 0.08 and a sample size of 10,000. A set of 100 datasets were simulated using the LD structure of the *CASP8* region containing 2871 fully genotyped SNPs. These were then reduced to contain 469 genotyped informative SNPs and the remaining 2402 SNPs were imputed.

All the results presented so far have been for datasets in which all SNPs of interest were genotyped. However, Figure 3 shows that when the causal SNP is one of many imputed SNPs, for the scenario considered, the results of filtering are similar to those when all SNPs are genotyped, provided an informative set of SNPs is genotyped. The ROC curves are displayed for LP and *p*-value filtering for a causal SNP with an OR of 1.1, an MAF of 0.13 and a sample size of 10,000 as these were the best performing filters with fully genotyped data. For both these filtering methods, the ROC curves for the partially imputed datasets are very similar to those for the fully genotyped datasets (the AUCs agree to two decimal places). Imputation in other scenarios was also examined and the agreement between the imputed and nonimputed analyses was similarly close. Therefore, these filtering methods also seem suitable for use with appropriately imputed genotype doses.

## Discussion

We have carried out a thorough simulation study to compare the performance of several easily computed univariate statistics with the aim of filtering SNPs in order to reduce the number to take forward for further analysis. Some of these methods have been previously used, the application of others as a filter is novel to the best of our knowledge. Our study focuses on small effect sizes and relatively rare SNPs. The results show that likelihood and *p*-value-based methods can be used to effectively filter candidate causal variants in fine-mapping studies for the scenarios we consider. We recommend using the LP method as this is generally the most efficacious. We carried out simulations based on three carefully chosen regions of the genome to reflect different local LD patterns. Despite being so different, LP filtering for causal SNPs with the same OR and MAF resulted in quite similar true and mean FPRs, meaning that our results might be applicable to many genomic regions under consideration in fine-mapping studies. We have also shown that genotype data which are partially imputed can also be filtered effectively using these methods. This conclusion relies on a set of carefully chosen informative SNPs being genotyped and expected genotype doses for the remaining SNPs being imputed using impute2 (Marchini & Howie, 2010).

In fine-mapping studies, investigators should choose the filter threshold based on the sample size and the estimated MAF and OR of the causal SNP (this can be estimated by fitting the individual logistic regression models to each of the SNPs and using the maximum fitted OR). The MAF of the causal SNP is not so easily estimated but crucially affects the effectiveness of LP filtering (Fig. 2C). For MAFs of 0.05 or less, filters might fail to capture the causal SNP with a high probability even with a sample size of 50,000 (data not shown). We suggest performing simulations for different MAF SNPs in the region of interest. Using a more lenient filtering threshold increases the probability of retaining the true causal SNP, but also captures more SNPs in total (Fig. 2B). With LP filtering, the proportion of SNPs that will be retained in total is fixed and, with a large number of SNPs being fine-mapped, this is approximately the same as the FPR and so should be chosen with this in mind.

These results also highlight the importance of using large sample sizes for fine-mapping and could be used as a reference before the genotyping stage of a study to aid in the decision of a minimum sample size. The required sample size to achieve any given power to “discriminate between highly correlated SNPs” at genome-wide levels of significance using RL has also been investigated in detail in Udler et al. (2010b). They have developed an online tool to calculate these sample sizes given other known information. So, filtering at a threshold of RL=1/100, with a causal SNP with an MAF of 0.12 and OR of 1.12, a sample size of 46,000 would be required to achieve a power of 0.9 if this causal SNP was in LD at *r*^2^ = 0.4 with SNP_*max*_ (the SNP with the largest likelihood). However, if the value of *r*^2^ between these two SNPs was 0.7, the sample size would need to be 92,000. This larger sample size is due to the difficultly to differentiate between the causal SNP and SNP_*max*_ when they are in such high LD.

Although using RL filtering with a threshold of 1/100 works well with a sample size of 20,000 when the effect size is moderate, as was the case at both the *FGFR2* and the *16q12* loci (Udler et al., 2009, 2010a), the effectiveness of this technique was seen to drop rapidly as the per-allele OR drops below 1.2. One of the major downfalls of using RL filters is the large amount of variation in FPR. This results in high uncertainty about the number of SNPs that will be retained after filtering. This is particularly a problem for causal SNPs with a low OR or MAF or when the sample size is small. LP filtering ensures that there is no uncertainty in the number of SNPs retained which is particularly useful when the number of SNPs that can be followed up is strictly limited.

The filters based on the structural relationships between variants did not produce encouraging results for causal SNPs with low ORs and MAFs. We showed that filtering in such scenarios using the PLD score developed by Zhu et al. (2012) is only slightly more efficacious than the more basic LD methods and did not perform as well as the LP filter. More work is needed to assess the utility of this method in other scenarios before firm conclusions can be drawn.

The competing outcomes of these methods are the probability of retaining the true causal SNP (TPR) and proportion of SNPs retained (FPR). A Bayesian decision theory approach has been developed by Wakefield (2007) to help deal with these two quantities. However, the difficultly with this method is the specification of a ratio of the cost of false nondiscovery to the cost of false discovery which many investigators might struggle to quantify with confidence.

The methods investigated in this study may be used when it is believed that a single variant is causing an association in a particular region of the genome. However, this may not be the case in many genomic regions. Several studies have also been carried out into alternative methods that may be more appropriate in identifying multiple causal variants in a single region, which is a hypothesis that many investigators are beginning to consider. For example, Vignal et al. (2011) demonstrated that penalised logistic regression (using HyperLASSO) was an effective method for analysing fine-mapping data from the *HLA* region for Rheumatoid Arthritis, and in general appears to be useful for finding multiple associations in a region of high LD.

Whether there are single or multiple causal variants in a region, causal SNP resolution may be improved by including information other than the genotype data. For example, there is now much data freely available on features of individual genetic variants in online databases such as the Encyclopaedia of DNA Elements (Encode Project Consortium, 2011). This includes features such as how conserved variants are across species and whether they are nonsynonymous. Bayesian methods of statistical analysis can be used to combine prior information about the likely functional role of an SNP with evidence from the genotype data and are a promising and exciting avenue of future research. Such methods include BLVMs (Fridley et al., 2011), stratified false discovery rates (Sun et al., 2006; Schork et al., 2013) and Bayes Factors (Wakefield, 2009; Knight et al., 2011).
